# Cryptotanshinone Suppresses BVDV Propagation by Suppressing Cell Apoptosis and Restoring Hormone Secretion in Bovine Granulosa Cells

**DOI:** 10.3390/v17111433

**Published:** 2025-10-28

**Authors:** Xiaoliang Chen, Haipeng Feng, Lei Wang, Jingyan Zhang, Xiaorong Lu, Guowei Xu, Siqi Liu, Qinxin Yang, Xiaowei Feng, Junyan Wang, Kang Zhang, Jianxi Li

**Affiliations:** 1 Technology Innovation Center of Traditional Chinese Veterinary Medicine of Gansu Province, Lanzhou Institute of Husbandry and Pharmaceutical Sciences of CAAS, Lanzhou 730050, China; 18148345073@163.com (X.C.); fenghp105@126.com (H.F.); wanglei03@caas.cn (L.W.); zwzh1223@126.com (J.Z.); luxr1993@163.com (X.L.); xuguowei@caas.cn (G.X.); liusiqi231004@163.com (S.L.); 15237309767@163.com (Q.Y.); 82101231377@caas.cn (X.F.); wjy620422@163.com (J.W.); 2 College of Veterinary Medicine, Hebei Agricultural University, Baoding 071000, China; 3 College of Veterinary Medicine, Gansu Agricultural University, Lanzhou 730070, China

**Keywords:** cryptotanshinone, bovine viral diarrhea virus (BVDV), bovine granulosa cells, anti-inflammation, hormone secretion

## Abstract

Bovine viral diarrhea virus (BVDV) constitutes a significant pathogen adversely threatening reproductive performance in the cattle industry, primarily by inducing ovarian dysfunction characterized by aberrant hormone synthesis and impaired follicular development. Although several commercial vaccines are available, they are insufficient for prevention and control BVDV infection, underscoring the necessity for the development of novel therapeutic drugs. This study aimed to investigate the antiviral activity of cryptotanshinone (CRY) against BVDV infection and its protective effects on bovine ovarian granulosa cells (BOGCs). An in vitro infection model was established by exposing BOGCs to BVDV. The results demonstrated that CRY exhibits anti-BVDV activity and alleviates detrimental effects on BOGCs through multiple mechanisms. Comparative analysis revealed that therapeutic administration of CRY constitutes the most effective mode of intervention. Furthermore, CRY promotes the secretion of estradiol (E_2_) and progesterone (P_4_) in BOGCs, counteracting the BVDV-induced reduction in these hormones. Moreover, CRY shows protective activity by mitigating BVDV-induced apoptosis in BOGCs. In summary, this study is the first to elucidate the inhibitory effect of CRY on BVDV and its regulatory role in BOGCs function, suggesting that CRY holds potential application value in the clinical treatment of BVDV-related reproductive disorders.

## 1. Introduction

Bovine viral diarrhea-mucosal disease (BVD) is a prevalent and reemerging infectious condition in cattle, caused by bovine viral diarrhea virus (BVDV). This disease commonly results in abortion, reproductive disorders, persistent infection, immunosuppression, and fatal mucosal lesions [1]. In clinical settings, the cattle infected with Bovine Viral Diarrhea (BVD) typically exhibit symptoms characterized by fever, severe diarrhea, mucosal erosion and ulcers, as well as abortion or stillbirth in pregnant cows [2]. A significant and enduring consequence of BVDV infection is the induction of diarrhea-associated immune tolerance and persistent infection-induced immunosuppression, which may persist for several weeks to months following infection and frequently culminate in mortality [3]. Over the past decade, BVDV has disseminated globally, infecting millions of bovines. The prevalence among infected bovine populations ranges Between 28% and 66% of cattle herds (populations) had at least one infected animal and in those herds that were infected, 40% to 90% of the individual animals were infected [4]. Consequently, BVDV imposes substantial morbidity and economic burdens in China and other affected countries, especially given the absence of effective vaccines or antiviral therapies. As a result, BVD represents one of the most economically impactful diseases in the global cattle industry. Annual economic losses from BVDV vary significantly, with global losses estimated in the billions of dollars [5].

Based on the new taxonomy released in 2024. Classification of the International Committee on Taxonomy of Viruses (https://ictv.global/report/chapter/flaviviridaeport/flaviviridaeport/flaviviridae/pestivirus, accessed on 15 October 2025), genus *Pestivirus* is composed of 19 recognized species, namely Pestivirus A (Bovine viral diarrhea virus 1, BVDV-1), Pestivirus B (Bovine viral diarrhea virus 2, BVDV-2), Pestivirus C (Classical swine fever virus, CSFV), Pestivirus D (Border disease virus, BDV), Pestivirus E (pronghorn antelope pestivirus), Pestivirus F (porcine pestivirus), Pestivirus G (giraffe pestivirus), Pestivirus H (Hobi-like pestivirus, HoBiPeV), Pestivirus I (Aydin-like pestivirus), Pestivirus J (rat pestivirus), Pestivirus K (atypical porcine pestivirus), Pestivirus L (Linda virus). Pestivirus M (Phccoena pestivirus), Pestivirus N (Tunisian sheep-like pestivirus), Pestivirus O (ovine/IT pestivirus), Pestivirus P (pangolin pestivirus), Pestivirus Q (rodent pestivirus), Pestivirus R (rodent pestivirus), Pestivirus S (bat pestivirus). Currently. BVDV is classified within genus *Pestivirus* of the family *Flaviviridae* and is characterized as a positive-sense single-stranded RNA virus. Its genome spans approximately 12.3 kb and contains a single large open reading frame [6,7]. Since its initial identification in United States in 1946 as a causative agent of reproductive losses in cattle, numerous studies have demonstrated that BVDV particles can persist in the ovaries for several weeks, leading to a range of reproductive pathologies such as ovarian inflammation, stalled follicular development and ovulatory dysfunction [8]. BVDV has become widespread in multiple countries and regions globally [9,10]. Due to the high genetic diversity and frequent mutations among viral strains, traditional attenuated live vaccines and inactivated vaccines have shown suboptimal efficacy in clinical prevention and control of this disease [11]. Consequently, there is an urgent need to develop highly effective antiviral drugs for the prevention and treatment of BVDV infection.

The ovaries are a principal target organ for BVDV infection. Studies indicate that BVDV can adversely affect bovine fertility and contributes to the generation of persistently infected individuals, wherein the virus can establish prolonged or chronic infections in ovarian or testicular tissues even following resolution of the acute phase [12]. By disrupting the physiological and endocrine functions of reproductive organs, BVDV markedly compromises normal ovarian function and reproductive performance. Specifically, BVDV infection can provoke ovarian inflammation, arrest of the estrous cycle, necrosis of granulosa cell, and delayed follicular development, resulting in suppressing estradiol (E_2_) and progesterone (P_4_) secretion, ultimately impairing estrus and ovulation processes [1]. Granulosa cells, as essential somatic components of the follicle, play irreplaceable roles in supporting oocyte development, synthesizing steroids, and follicular maturation. Their apoptosis or dysfunction directly precipitates aberrant follicular development [13]. Considering the critical importance of ovarian function in bovine reproduction and the substantial economic losses incurred by the livestock industry due to BVDV-related ovarian pathology, developing effective intervention strategies to mitigate BVDV-induced ovarian damage is of considerable scientific significance and practical promise.

Cryptotanshinone (CRY) is a diterpenoid quinone compound extracted from the traditional Chinese medicinal herb *Salvia miltiorrhiza*. It has been demonstrated to show multiple bio-activities, including anti-breast cancer effects [14], anti-inflammatory properties [15], antioxidant stress protection, and anti-apoptotic activity [16]. Intriguingly, CRY has been reported to mitigate condition such as polycystic ovary syndrome, premature ovarian failure, and ovarian damage, as well regulating hormone secretion and restoring ovarian function [17,18,19]. Recent studies have revealed that CRY exhibits significant antiviral effects against the novel coronavirus and porcine reproductive and respiratory syndrome virus [20,21]. Nevertheless, its inhibitory effect on BVDV have yet to be elucidated. Accordingly, the present study aims to explore, for the first time, the potential of CRY as a therapeutic drug against BVDV. Utilizing a BVDV-infected BOGCs model, we evaluated the effects of CRY on viral replication, inflammatory response, hormone secretion, and apoptosis. This research seeks to elucidate the antiviral mechanisms of CRY and to contribute novel strategies for clinical prevention and management of BVDV infection.

## 2. Materials and Methods

### 2.1. In Vitro Culture and Identification of Bovine Ovarian Granulosa Cells

Fresh ovaries were collected from healthy Holstein dairy cows at a slaughterhouse in Wuzhong City, Ningxia Hui Autonomous Region. They were placed in a thermally insulated bucket filled with normal saline solution with 100 IU/mL penicillin-streptomycin, maintained at 30–37 °C, and transported to the laboratory within 2 h. Following our Lab’s established protocol, medium-sized follicles (5–8 mm diameter) were harvested using a 5 mL syringe (26 G needle). Specifically, healthy follicles within the 5–8 mm range were rinsed three times with sterile PBS, then washed with serum-free medium. Bovine ovarian granulosa cells (BOGCs) were incubated in DMEM/F12 medium (Gibco) contained with 1% penicillin/streptomycin (Gibco) and 10% Fetal bovine serum (FBS) (Gibco), kept at 37 °C in a 5% CO_2_ incubator. After being centrifugated, BOGCs were collected, and washed three times with culture medium, and finally seeded into T25 flasks [22,23].

### 2.2. Virus Amplification

Madin-Darby bovine kidney (MDBK) cells and the BVDV NADL standard strain (CP type) (CVCC AV67) were purchased from the China Veterinary Drug Control Institute. MDBK cells were cultured under the same conditions as described of reference [24], employing RPMI 1640 medium with the incorporation of 1% penicillin/streptomycin and 10% FBS. After being amplified NADL BVDV in MDBK cells, the infected cells with culture medium were subjected to three cycles of freezing and thawing. Subsequently, the mixture was centrifuged at 1000 r/min for 10 min, and the supernatant was collected as the viral stock solution.

### 2.3. Virulence Assay

A density of 1 × 10^4^ BOGCs per well were seeded in a 96-well plate and incubated at 37 °C for 24 h. The virus was performed in a 10-fold serial dilution and inoculated into the cells, followed by continued incubation at 37 °C. The cells were observed continuously for 3–5 days, recording the cytopathic effect (CPE) in each well. The viral titer was calculated using the Reed-Muench method.

### 2.4. Cell Viability Assay

After being dissolved in Dimethyl sulfoxide (DMSO), 1 mM stock solution of CRY (China National Institutes for Drug Control, Beijing, China, Catalog No.: 110852-201807) was prepared using serum-free DMEM/F12 medium. The tested concentrations of CRY were prepared using same medium. BOGCs were seeded at a density of 1 × 10^4^ cells per well in a 96-well plate, and incubating at 37 °C for 24 h. After then, the different concentrations of CRY was added and continued incubation for more 24 h. Subsequently, the previous medium was discarded, the cells were washed once with PBS, then mixed serum-free medium of 100 μL with Cell Counting Kit-8 (CCK-8) reagent of 10 μL (Biosharp, Hefei, China), and incubated at 37 °C in the dark for 2 h. The optical density (OD) was measured at 450 nm using a microplate reader (BioTek, Winooski, VT, USA).

Cell viability was calculated using the following Equation (1):

Cell Viability (%) = (Experimental Group OD Value − Blank Group OD Value)/(Control Group OD Value − Blank Group OD Value) × 100%(1)

Notes:

Experimental Group: Treatment groups containing different concentrations of CRY

Control Group: Negative control group without CRY

Blank Group: Blank control group containing only medium and DMSO.

### 2.5. Trials of CRY’s Effect on BVDV

Prevention: To investigate the preventive effect of CRY against BVDV, BOGCs were treated with CRY of designed concentrations for 2 h. The supernatant was discarded, BVDV was added, and the mixture was incubated for 2 h. The supernatant was discarded again, and cells were cultured in medium for 24 h before samples were collected.

Neutralization: To evaluate the neutralizing effect of CRY on BVDV, the drug and BVDV were mixed at a 1:1 ratio and incubated at 37 °C for 2 h. The mixture was then added into cells and incubated for 24 h before collecting samples.

Therapeutic Effect: To determine the therapeutic effect of CRY on BVDV, cells were firstly infected with BVDV for 2 h. The supernatant was discarded, CRY was added, and cells with the mixture were incubated for 24 h. Then, samples were collected.

Adsorption: To examine the inhibition of CRY on BVDV adsorption, cells were treated with CRY at 4 °C for 2 h. The supernatant was discarded, cells were infected BVDV that had been pre-incubated with CRY and incubated at 37 °C for 2 h. Then the supernatant was discarded, and cells were cultured at 37 °C for 24 h in the medium. Then samples were collected.

Internalization: To examine the inhibition of CRY on BVDV internalization, BVDV was incubated with BOGCs at 4 °C for 2 h. The supernatant was discarded, CRY was added, and cells were incubated at 37 °C for 24 h before collecting samples.

### 2.6. Immunofluorescence

The BOGCs pre-treated with CRY or BVDV were washed three times with PBS for 5 min each. The cells were fixed with 4% paraformaldehyde for 30 min at room temperature. Then, they were treated with 0.1% Triton X-100 (Beyotime, Shanghai, China) for 5 min, then washed three times for 5 min each. They were blocked with a surface-leaf staining blocking solution for 1 h, then followed by three times washes with PBS for 5 min each. Cells were incubated overnight with primary antibodies against FSHR (Abcam, Shanghai, China, ab113421), CYP19A1 (Abclonal, Wuhan, China, A12238), and BVDV (Santa Cruz Biotechnology, Santa Cruz, CA, USA, sc-101592). Then, cells were incubated with goat anti-mouse secondary antibody and goat anti-rabbit secondary antibody, respectively. Subsequently, cells were mounted with anti-fluorescent mounting medium containing DAPI. Finally, positive signals were observed under a laser confocal microscope (LMS800, ZEISS, Oberkochen, Germany).

### 2.7. Gene Relative Expression and Absolute Quantitation

Viral RNA was isolated from collected specimens using a viral RNA/DNA extraction kit (Accurate, Changsha, China). Absolute quantification of the viral RNA was achieved through a standard curve using constructed recombinant plasmid templates. The primers pair utilized in this study was designed in-house, including the forward primer BVDV-F (5′-GGCATGCCCTTAGTAGGACT-3′) and the reverse primer BVDV-R (5′-GCCATGTACAGCAGACAT-3′). The RT-qPCR procedure included a reverse transcription step at 42 °C for 5 min and an initial denaturation at 95 °C for 30 s, followed by 40 cycles consisting of denaturation at 95 °C for 5 s and annealing/extension at 60 °C for 30 s. For the analysis of cellular RNA, total RNA was extracted from cultured cells using the Trizol reagent. The purity and concentration of the extracted RNA were evaluated using a NanoDrop ND-2000 spectrophotometer (Thermo Fisher Scientific, Waltham, MA, USA). Complementary DNA (cDNA) was synthesized from the RNA samples using the Revert Aid First-Strand cDNA Synthesis Kit (Accurate, Changsha, China). Gene-specific primers were designed using Primer-BLAST software (BLAST+ 2.17.0) (refer to Table 1). Real-time PCR was conducted on either a LightCycler 480 system (Roche, Laval, Quebec, Canada) or an Applied Biosystems 7500 Real-Time PCR System (ABI, Los Angeles, CA, USA), with SYBR^®^ Green Premix Pro Taq HS qPCR Reagent Kit (Rox Plus) (Accurate, Changsha, China). The amplification program consisted of an initial denaturation at 95 °C for 30 s, followed by 40 cycles of denaturation at 95 °C for 5 s and extension at 60 °C for 30 s. Relative mRNA expression levels were analyzed using the 2^−ΔΔCT^ method.

### 2.8. Measurement of Proinflammatory Cytokine Secretion Levels

Following cell culture, the supernatant was harvested and centrifuged at 3000× *g* for 10 min to eliminate cellular debris. The concentrations of proinflammatory cytokines, including interleukin-6 (IL-6), IL-1β, and tumor necrosis factor-α (TNF-α) were measured using commercially available ELISA kits (MLBio, Shanghai, China) according to the manufacturer’s guidelines. All samples and standards were analyzed in duplicate. The absorbance of each well was read at a wavelength of 450 nm with a microplate reader (BioTek Instruments, Winooski, VT, USA). The levels of the respective cytokines were calculated by referencing the standard curves established for each cytokine.

### 2.9. Estradiol and Progesterone Secretion Level Assay

The concentrations of E_2_ and P_4_ in supernatant of cells were measured using commercially available ELISA kits (MMbio Nanjing, Jiangsu, China), in accordance with the manufacturer’s protocol. All samples and standards were run in duplicate. The absorbance of each well was recorded at 450 nm using a microplate reader (BioTek Instruments, Winooski, VT, USA). Hormone levels were calculated based on the standard curves established for each specific assay.

### 2.10. Apoptosis Analysis

Cells were grown in 6-well plates until reaching approximately 80% confluence. Following a 2 h exposure to 100 TCID_50_ of BVDV, the culture supernatant was removed. The cells were then incubated with low, medium, and high concentrations of CRY. Twenty-four hours later, the extent of apoptosis was assessed using the Annexin V-FITC/PI Apoptosis Detection Kit (4A BIOTECH, Suzhou, Jiangsu, China) through flow cytometric analysis (Beckman Coulter, Brea, CA, USA).

### 2.11. Western Blotting

Cells were seeded in 6-well plates and cultured until reaching approximately 80% confluence. Following a 2-h exposure to 100 TCID_50_ of BVDV, the supernatant was removed. The cells were then exposed to low, medium, and high concentrations of CRY. After 24 h, cell lysates were prepared using a protein lysis buffer (Beyotime, Shanghai, China) for protein extraction. Proteins were resolved on a 10% SDS-PAGE gel and subsequently transferred onto a PVDF membrane. The membrane was blocked and then incubated overnight at 4 °C with primary antibodies targeting BCL-2, BAX, and β-actin. A secondary goat anti-mouse antibody was applied for a duration of 1 h. Immunoreactive bands were visualized using an ECL chemiluminescence substrate and captured by imaging. The intensity of the bands was quantified using ImageJ software (1.54p), with β-actin serving as the internal reference.

### 2.12. Data Analysis

Data were analyzed and visualized using GraphPad Prism 9.0 software through one-way ANOVA. The results are presented as mean ± standard error of the mean (SEM). *p* value less than 0.05 was considered statistically significant. All samples were analyzed in triplicate.

## 3. Results

### 3.1. Bovine Granulosa Cells Identification

**Figure 1:**
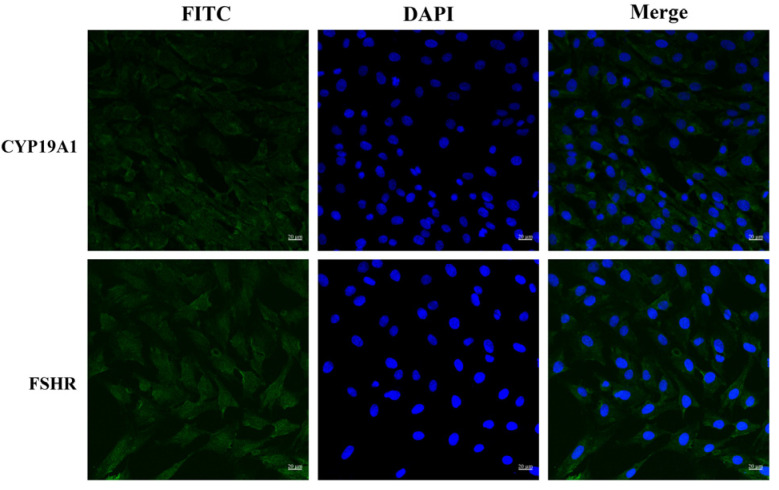
Immunofluorescence assay of BOGCs stained with antibodies against CYP19A1 and FSHR. Green fluorescence represents the presence of CYP19A1 or FSHR, while blue fluorescence indicates the position of the cell nuclei. Scale bar = 20 μM.

BOGCs were aseptically collected from bovine ovaries sourced from a local slaughterhouse. The identity and purity of the isolated BOGCs were verified using immunofluorescence staining against two specific markers: follicle-stimulating hormone receptor (FSHR) and cytochrome P450 family 19 subfamily A member 1 (CYP19A1), an essential enzyme involved in estrogen biosynthesis [25]. As shown in Figure 1, the immunofluorescence analysis revealed robust positive signals for both FSHR and CYP19A1 in the majority of the cell population. The result demonstrated that the isolated cells were highly enriched and retained their functional properties. Such a degree of purity supports the suitability of the BOGCs for subsequent experimental investigations.

### 3.2. The Optimal Concentration of CRY

**Figure 2:**
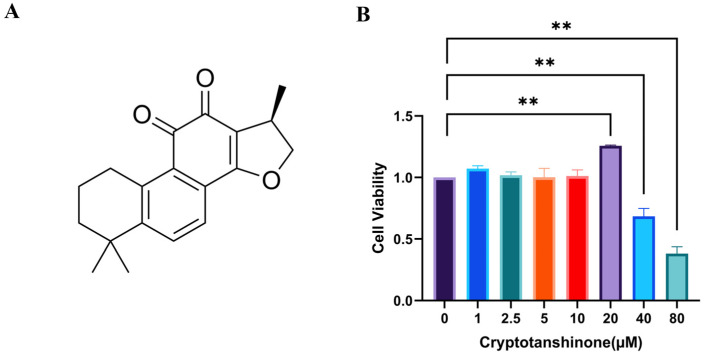
(**A**) Structure of CRY; (**B**) Effect of cryptotanshinone (CRY) on the viability of BOGCs for 24 h. *p* value less than 0.05 was considered statistically significant. All samples were analyzed in triplicate. The data presented herein are the mean ± standard error of three replicate experiments. The symbol ** indicates a significance level of *p* < 0.01.

CRY has been previously documented to possess notable antiviral activity. To determine a concentration range that is both non-cytotoxic and functionally efficacious for subsequent antiviral experiments, the effect of CRY on BOGCs viability was assessed. As shown in Figure 2, treatment with CRY at concentrations of 40 μM and 80 μM resulted in a substantial reduction in metabolic activity of BOGCs, suggesting pronounced cytotoxicity. Consequently, to preserve cellular viability while potentially retaining antiviral activity, the concentrations of CRY at 5 μM, 10 μM, and 20 μM were chosen for further experimental studies.

### 3.3. CRY Inhibits BVDV Replication in Three Treatment Methods

**Figure 3:**
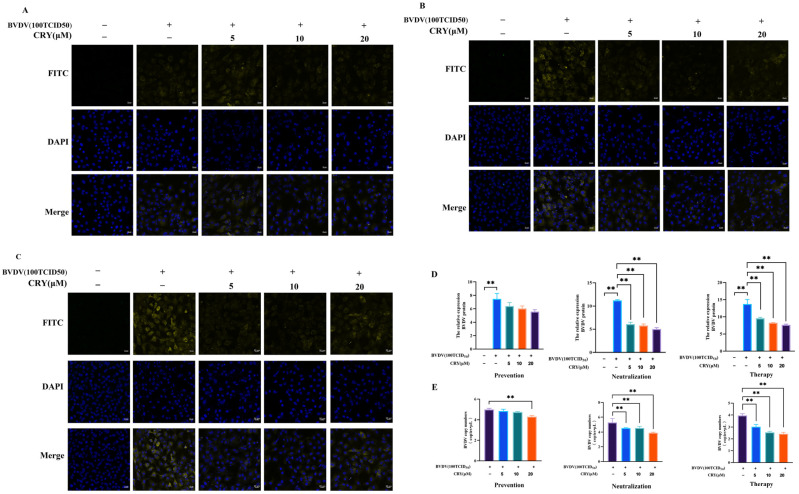
CRY inhibits the replication of BVDV on BOGCs through prevention, neutralization, and therapy methods. (**A**) Immunofluorescence analysis of BVDV expression in the prevention group. (**B**) Detection of BVDV expression by immunofluorescence in the neutralization group. (**C**) Immunofluorescence evaluation of BVDV expression levels in the therapy group. (**D**) Proteins levels of BVDV of (**A**–**C**) by image J analysis. (**E**) BVDV nucleic acid copies number was detected by RT-qPCR. The data presented herein are the mean ± standard error of triplicate. The data presented herein are the mean ± standard error of three replicate experiments. The symbol ** indicates a significance level of *p* < 0.01.

BOGCs were infected by BVDV after 24 h, which can be exhibited a marked cytopathic effect (CPE), with a TCID_50_ value of 10^−4.84^/0.1 mL (Figure S1). To elucidate the multifaceted antiviral actions of CRY in ovarian granulosa cells, five experimental groups were established, including control group and BVDV group, and BVDV+ 5 μM CRY group, BVDV+ 10 μM CRY group, BVDV+ 20 μM CRY group. Each group represented a unique intervention strategy (Figure 3). The cells pretreated with a high dose of CRY for 2 h exhibited a significant reduction in BVDV mRNA expression. Conversely, lower and intermediate doses did not produce a statistically significant inhibitory effect at the mRNA level. Protein analysis revealed a consistent downward trend in BVDV expression across all dose groups. However, these reductions did not reach statistical significance (Figure 3A,D). When CRY at varying concentrations was mixed with 100 TCID_50_ BVDV at 1:1 ratio and co-cultured for 2 h, dose-dependent suppression of BVDV mRNA expression was observed in the granulosa cells, with significant suppression evident at all tested concentrations. Correspondingly, BVDV protein level was markedly reduced, corroborating the virus-neutralizing capacity of CRY. Furthermore, following a 2h infection with 100 TCID_50_ of BVDV, subsequent treatment with CRY and continuous culture for 24 h resulted in a pronounced, dose-dependent decrease in BVDV mRNA levels (Figure 3B,D). Protein expression mirrored this trend, aligning with the mRNA findings. Collectively, these results indicate that CRY exerts a potent inhibitory effect on both the mRNA and protein expression levels of BVDV in infected cells, highlighting its therapeutic potential against established BVDV infection (Figure 3C,D).

### 3.4. Effects of CRY on BVDV Adsorption and Internalization in BOGCs

**Figure 4:**
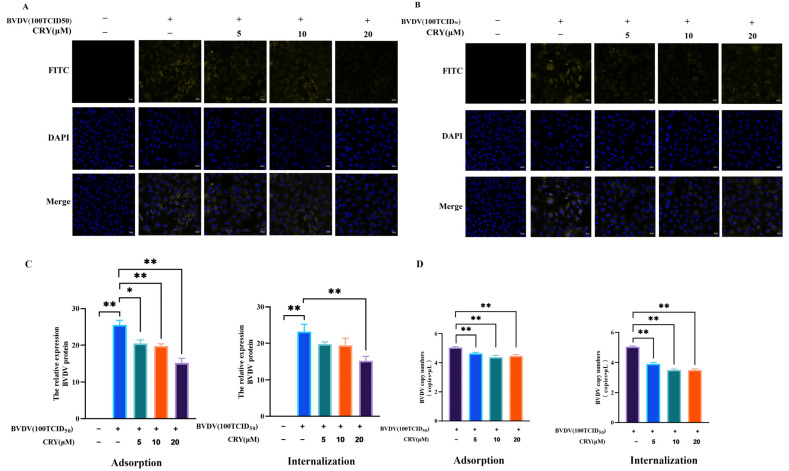
CRY blocks the replication of BVDV on BOGCs through adsorption and internalization. (**A**) Immunofluorescence of BVDV levels in the adsorption group. (**B**) Immunofluorescence of BVDV levels in the internalization group. (**C**) As illustrated in (**A**,**B**), the Image J statistics of BVDV proteins levels. (**D**) BVDV nucleic acid copy numbers. The data presented herein are the mean ± standard error of three replicate experiments. The symbols * and ** indicate significance levels of *p* < 0.05 and *p* < 0.01 respectively.

To explore the impact of CRY on the adsorption of BVDV, cells were exposed to concentrations ranging from 5 to 20 μM of CRY at 4 °C for a duration of 2 h. After removal of the supernatant, the cells were subjected to infect with BVDV pre-treated with CRY and maintained under incubation for at 37 °C another 2 h. Subsequently, the supernatant was discarded, and the cells were cultivated in fresh medium for a duration of 37 °C for 24 h. As shown in Figure 4A,C,D, samples collected at the end of incubation period underwent further analysis. Notably, both the mRNA and protein expression levels of BVDV in the groups treated with CRY were markedly lower than those observed in the untreated control group. These results indicated that CRY exerts a considerable inhibitory influence on BVDV adsorption to granulosa cells, with the degree of inhibition positively correlating to the applied CRY concentration.

To assess the effect of CRY on BVDV internalization, BVDV was first allowed to bind to granulosa cells at 4 °C for 2 h. Following removal of the supernatant, CRY was introduced to the cells, which were then incubated for an additional at 37 °C for 24 h. As shown in Figure 4B–D, the mRNA and protein expression levels of BVDV in the CRY-treated cells were markedly reduced in comparison to the control group. BVDV nucleic acid levels were reduced in cells treated with CRY, whereas BVDV protein expression was higher than that in the BVDV group only in the 20 μM treatment group. These results suggest that CRY exhibits a significant inhibitory effect on the internalization of BVDV into granulosa cells, with the extent of inhibition on mRNA expression demonstrating a dose-dependent fashion.

### 3.5. CRY Inhibits BVDV-Induced Inflammatory Responses

**Figure 5:**
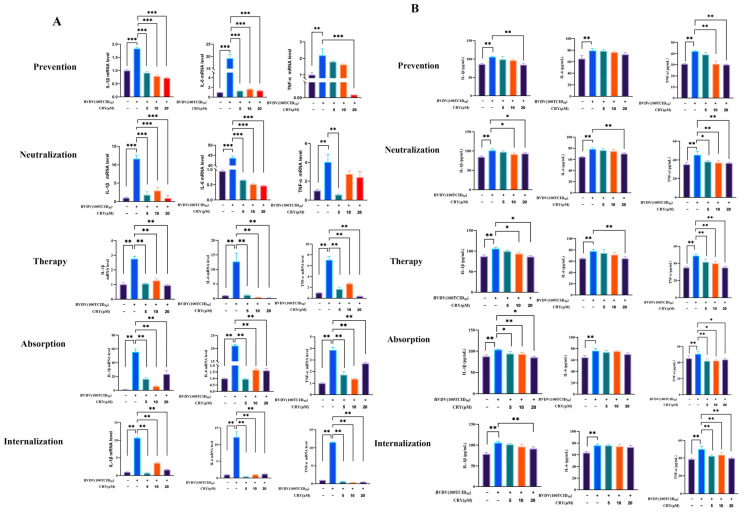
CRY inhibits the inflammatory reactions of BVDV on BOGCs. (**A**) The relative expression levels of IL-1β, IL-6, and TNF-α mRNA. (**B**) The protein expression levels of IL-1β, IL-6, and TNF-α. * indicates *p* < 0.05, ** indicates *p* < 0.01, *** indicates *p* < 0.001.

To further elucidate the extensive anti-inflammatory effects of CRY in the context of BVDV-induced inflammation in ovarian granulosa cells, the expression levels of key inflammatory cytokines (IL-6, IL-1β, and TNF-α) were analyzed at both the mRNA and protein levels (Figure 5). Compared to the BVDV-infected group, treatment with CRY at 5 μM, 10 μM, and 20 μM significantly downregulated the mRNA high expression of IL-6 and IL-1β across multiple experimental conditions, including the pre-treatment, neutralization, therapeutic treatment, and viral adsorption/internalization inhibition groups. However, the mRNA levels of TNF-α were also markedly reduced specifically in the therapeutic treatment groups. At the protein level, all tested concentrations of CRY significantly suppressed TNF-α secretion in both the therapeutic treatment group and the groups subjected to viral adsorption and internalization inhibition, whereas IL-1β and IL-6 protein levels no significant reduction was observed compared to the BVDV-infected control group. Collectively, these findings indicate that CRY effectively mitigates inflammation associated with BVDV infection and its subsequent viral replication.

### 3.6. Effect of CRY on E_2_ and P_4_ Levels of BOGCs

**Figure 6:**
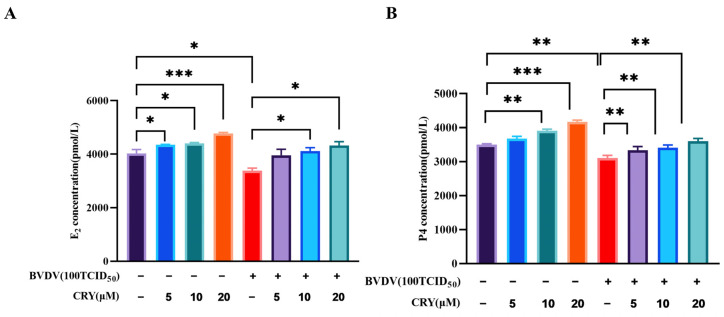
Effects of CRY on the levels of E_2_ and P_4_. (**A**) Effect of CRY on E_2_ expression levels; (**B**) Effect of CRY on P_4_ expression levels. * indicates *p* < 0.05 and ** indicates *p* < 0.01, *** indicates *p* < 0.001.

To examine the effect of CRY on E_2_ and P_4_ levels, BOGCs were treated with different doses of CRY. As shown in Figure 6, CRY at concentration ranging from 5 μM to 20 μM significantly promoted E_2_ and P_4_ secretion in a dose-dependent manner. Following exposure to BVDV at 100TCID_50_, E_2_ and P_4_ level was decreased significantly. However, treatment with 10 μM and 20 μM CRY significantly increased the E_2_ and P_4_ level caused by BVDV (*p* < 0.05). These finding revealed that CRY can reverse the dysregulated secretion of E_2_ and P_4_ for BVDV infection in BOGCs.

### 3.7. Effects of CRY on Apoptosis Induced by BVDV in BOGCs

**Figure 7:**
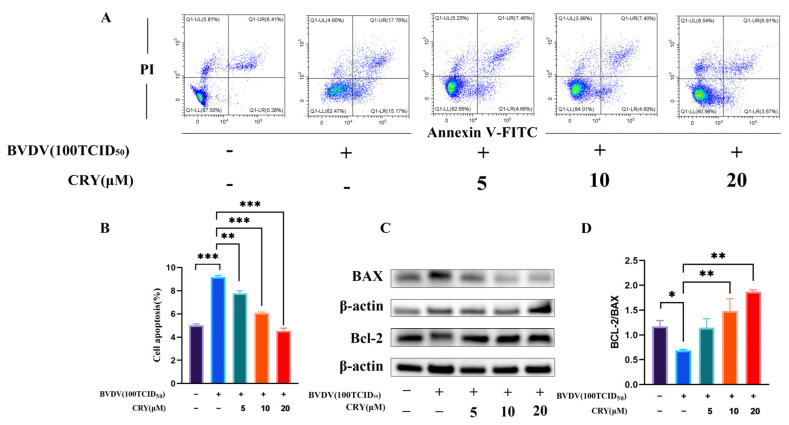
CRY alleviates BVDV-induced apoptosis in BOGCs. (**A**) Apoptosis levels were quantified by flow cytometric analysis. (**B**) Statistical analysis of apoptosis rates based on the results presented in Panel (**A**). Protein expression levels of BAX and Bcl-2. (**D**) The ratio of BCL-2 to BAX was determined by Image J for (**C**). * indicates *p* < 0.05 and ** indicates *p* < 0.01, *** indicates *p* < 0.001.

To evaluate the potential anti-apoptotic effects of CRY in the context of BVDV infection, an experimental model of granular cells infected with BVDV was established. CRY was administered at concentrations of 5 μM, 10 μM, and 20 μM to the infected cells. The results of flow cytometric analysis indicated that BVDV infection significantly induced apoptosis in granular cells (Figure 7A). Conversely, treatment with CRY effectively attenuated apoptosis in a dose-dependent manner (Figure 7B). Compared to the BVDV-infected group, all CRY-treated groups showed considerable reduction in apoptotic damage, with the highest dose exhibiting the most pronounced protective outcome. Furthermore, as illustrated in Figure 7C,D, the Bcl-2/BAX ratio in the CRY-treated group was significantly higher compared to that in the infected group. These findings suggest that CRY possesses substantial anti-apoptotic properties against BVDV-induced cellular apoptosis.

## 4. Discussion

Bovine viral diarrhea virus (BVDV) is a critical pathogen imposing substantial economic burdens in the global cattle industry, primarily due to its involvement in reproductive disorders, immune dysfunction, and diarrhea diseases [26]. The virus exhibits a tropism for preferentially infects ovarian tissues, leading to disruptions in steroid hormone production, follicular development, and the establishment of persistent infections [1]. In present study, we elucidate for the first time that CRY, a bioactive compound derived from *Salvia miltiorrhiza*, demonstrates strong antiviral activity against BVDV in BOGCs. Our experimental data indicate that CRY exerts its effects via multiple mechanisms, including direct viral inactivation, inhibition of viral attachment and entry, and suppression of viral replication post internalization, thereby effectively reducing BVDV infection. Additionally, CRY markedly mitigated the inflammatory response triggered by BVDV, normalized aberrant hormone secretion, and reduced apoptosis in BOGCs. These findings highlight the potential of CRY as a candidate for the prevention and treatment of BVDV infections.

Currently, CRY may be the result of direct virucidal activity or as regulators of processes that occur within the host cells. The direct virucidal activity mechanism suggests that lipophilic may disrupt the viral envelope, thereby, damaging the structural integrity of the virus and rendering it incapable of infecting host cells. It is known that lipophilic compounds can interact with the lipid bilayer of membranes, which may lead to instability of the viral envelope. Previous studies have confirmed that certain lipophilic molecules can penetrate the viral membrane, triggering disintegration and loss of infectivity [27]. CRY may exert its antiviral effects through multiple pathways. Although the trends of mRNA expression and protein levels were consistent, some differences were not statistically significant, which could be attributed to the sensitivity of the detection methods or the mRNA levels not fully reflecting the viral inhibition. We speculate that CRY targets the RNA transcription stage of BVDV, not the protein synthesis stage. RNA transcription requires the participation of multiple replicase enzymes. Therefore, we speculate that CRY may target a nonstructural protease of BVDV to exert its effect, which is another antiviral pathway worthy of in-depth analysis. However, at the protein level, BVDV replication was significantly suppressed by CRY. In neutralization and therapeutic assays, CRY exhibited greater inhibition of BVDV mRNA and protein expression levels of BVDV in BOGCs than prophylactic treatment, indicating its capacity to directly inactivate BVDV and inhibit the viral replication in target cells, which suggesting that it may interact with viral surface proteins, thereby preventing viral attachment to host cells. Similar antiviral activities of CRY have been documented against SARS-CoV-2 and the arterivirus PRRSV [20,21]. This model of action parallels that of natural products such as gypenoside [28], phlorizin [29], native banana lectin [30], which interfere with receptor-mediated endocytosis during viral entry. Moreover, CRY significantly hindered both viral attachment and internalization, indicating its ability to interfere with early virus-host interactions. Consistent with these findings, a study has also shown that CRY exerts inhibitory effects on VSV, H1N1, EMCV, and HSV-1 both pre- and post-viral entry [31]. Notably, CRY also displayed therapeutic efficacy when administrated post-infection in the present study, demonstrating its capacity to inhibit intracellular viral replication. Recent research further corroborates CRY’s strong inhibition on viral replication following ZIKV entry into Vero cells [32]. Such multi-target antiviral action are highly advantages, as they target multiple stages of the viral life cycle and lower the likelihood of drug resistance, a common drawback of single-target therapies.

A salient finding of this study is the identification of CRY potent anti-inflammatory properties in BVDV-infected BOGCs. Usually, inflammatory response is a common pathological process after viral infection [33]. However, BVDV frequently establishes persistent infection in susceptible dairy cows, potentially leading to chronic inflammation injury. Clinically, BVDV-induced reproductive damage often manifests as ovarian inflammation, dominated by the augment of pro-IL-1β expression in BT cells and cleavage pro-IL-1β into IL-1β to activate the NF-κB pathway [34]. In the present study, BVDV infection significantly upregulated IL-1β, IL-6, and TNF-α production in both intracellular and extracellular. The overexpression of these cytokines at both the gene and protein levels induced by BVDV infection was significantly suppressed by CRY treatment. This result is consistent with the previously reported anti-inflammatory properties of CRY [35]. The research indicated that CRY alleviates BVDV-induced inflammatory damage to BOGCs by down-regulating the inflammatory cytokines release in BOGCs. Anti-inflammatory action of CRY may be attributed to the regulation of signaling cascades such as NF-κB and STAT3, which is frequently activated during viral infections and has been previously shown to be modulated by CRY in other pathological contexts [21,36,37]. By attenuating inflammation, CRY may preserve granulosa cell function and create a microenvironment less favorable for viral persistence.

Granulosa cells are essential for follicular development and the biosynthesis of steroid hormones [38]. BVDV infection disrupts endocrine function, resulting in decreased levels of E_2_ and P_4_, which are vital for maintaining estrous cycles and supporting pregnancy [1,39]. Our data demonstrated that CRY not only enhanced basal E_2_ and P_4_ secretion in uninfected cells but also restored hormone levels in infected cells. CRY can regulate the secretion disorder of E_2_ in other reproduction diseases, such as polycystic ovary syndrome [40] and benign prostatic hyperplasia progression [41]. Together, CRY exhibits a property of promotion to the secretion of E_2_ and P_4_ in bovine granulosa cells, thereby modulating steroid hormone function. The hormone-restorative capacity of CRY highlights its therapeutic potential in mitigating BVDV-induced reproductive dysfunction.

In addition, our results indicated that CRY significantly reduced apoptosis in BVDV-infected BOGCs. Flow cytometry analysis showed dose-dependent reduction in apoptosis cell, corroborated by an elevated Bcl-2/BAX ratio, a well-established indicator of mitochondrial apoptotic regulation [42]. While apoptosis is a host defense mechanism, BVDV is known to induce it, contributing to tissue damage. CRY’s anti-apoptotic effect here is beneficial as it preserves the vital granulosa cell population, which is central to the study’s therapeutic goal of maintaining ovarian function. BVDV is known to activate apoptotic pathways through mechanisms such as endoplasmic reticulum stress and death receptor signaling [43,44]. The anti-apoptotic effects of CRY have been validated in previous researches [45], paralleling the activity of other natural compounds, such as hyperoside [46], rhamnocitrin [47], and rosmarinic [48]. Anti-apoptotic effect is shown, although apoptosis is occasionally observed as part of host response to infection. Inhibition potentially predisposes to persistent. Nevertheless, our findings suggested that CRY preserve cellular viability and function through inhibiting viral replication and eliminate apoptotic cells, which is crucial for maintaining ovarian health and reproductive performance.

The implication of these findings extends beyond the control of BVDV. The diverse biological activities of CRY including antiviral, anti-inflammatory, and anti-apoptotic effects, which suggest its potential as a broad-spectrum therapeutic agent [49,50]. Earlier studies have documented its effectiveness against other viruses such as PRRSV and SARS-CoV-2, pointing to a possible common antiviral mechanism, potentially involving interference with viral entry or replication processes shared among various viruses [20,21]. Moreover, its beneficial effects on ovarian function are consistent with previous reports on its therapeutic role in conditions such as polycystic ovary syndrome and premature ovarian insufficiency [39,51].

Nonetheless, several aspects warrant further investigation. The precise molecular targets of CRY in the context of BVDV infection require further elucidate. Potential mechanisms may involve disruption of the viral envelope, inhibition of viral RNA-dependent RNA polymerase, or modulation of host factors such as viral receptors or components of the innate immune system. Future research should gradually advance the use of CRY in the clinical treatment of BVDV infection. A multidimensional evaluation system, including parameters such as viral clearance efficiency, recovery time for infected cattle, and improvement in clinical symptoms, will be employed to comprehensively assess its potential therapeutic effects and application feasibility in real-world clinical settings.

## 5. Conclusions

In summary, this study presents compelling evidence that CRY effectively inhibits BVDV through attenuating virus-induced inflammation and apoptosis, and restores hormonal balance in BOGCs. These findings position CRY as a promising natural compound for the prevention of BVDV infection and its associated reproductive complications. Further investigations utilizing animal models is warranted to validate these outcomes and to facilitate the development of CRY-based therapeutic interventions for clinical in veterinary medicine.

## Figures and Tables

**Table 1:** Primers used in this study.

Gene	Primer Sequence (5′-3′)	Product Length (bp)	Serial Number
IL-6	F: AGATCCTGAAGCAAAAGATCGC	101	NM_173923.2
	R: TGCGTTCTTTACCCACTCGT		
IL-1β	F: CCTCCGACGAGTTTCTGTGT	158	NM_174093.1
	R: GCTCATGCAGAACACCACTTC		
TNF-α	F: CCCACGTTGTAGCCGACAT	133	NM_173966.3
	R: ATGAGGTAAAGCCCGTCAGC		
β-actin	F: ATCGGCAATGAGCGGTTCC	143	NM_173979.3
	R: GTGTTGGCGTAGAGGTCCTTG		

## Data Availability

All data included in this study are available upon request by contact with the corresponding author.

## Supplementary Materials

The following supporting information can be down-loaded at: https://www.mdpi.com/article/10.3390/v17111433/s1, Figure S1. Effect of BVDV infection on the mor-phology of MDBK and BOGCs cells. (A) BVDV infection on the morphology of MDBK. (B) BVDV infection on the morphology of BOGCs. (C) The morphology of untreated MDBK cells. (D) The morphology of untreated BOGCs. Scale bar = 20 μM.

## Abbreviations

The following abbreviations are used in this manuscript:

BVDV	Bovine viral diarrhea virus
BVD	Bovine viral diarrhea-mucosal disease
CRY	Cryptotanshinone
BOGCs	Bovine ovarian granulosa cells
E_2_	Estradiol
P_4_	Progesterone
MDBK	Madin-Darby bovine kidney
CPE	Cytopathic effect
CP	Cytopathic Biotype
OD	Optical density
IL	Interleukin
TNF-α	Tumor necrosis factor-α
TCID_50_	50% Tissue culture infective dose
FSHR	Follicle-stimulating hormone receptor
CYP19A1	Cytochrome P450 family 19 subfamily A member 1
Bcl-2	B-cell lymphoma/lewkmia-2
BAX	BCL2-Associated X Protein
