# Supplementary material for: Cryptotanshinone Suppresses BVDV Propagation by Suppressing Cell Apoptosis and Restoring Hormone Secretion in Bovine Granulosa Cells
Source: Viruses. 2025 Oct 28;17(11):1433. doi: 10.3390/v17111433 (PMC12656771; doi:10.3390/v17111433)
Supplement: Supplementary file 1 [file viruses-17-01433-s001.zip › viruses-3921576-supplementary.pdf]

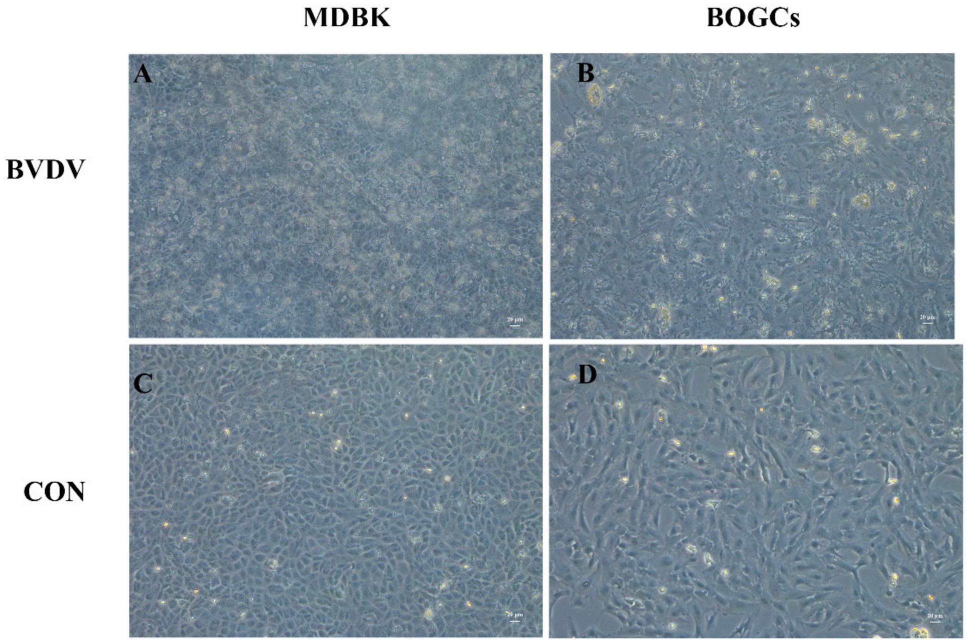

Figure S1. Effect of BVDV infection on the morphology of MDBK and BOGCs cells. (A) BVDV infection on the morphology of MDBK. (B) BVDV infection on the morphology of BOGCs. (C) The morphology of untreated MDBK cells. (D) The morphology of untreated BOGCs. Scale bar = 20 μM.
